# Supplementary material for: Rhythm of the Night (and Day): Predictive Metabolic Modeling of Diurnal Growth in Chlamydomonas
Source: mSystems. 2022 Jun 13;7(4):e00176-22. doi: 10.1128/msystems.00176-22 (PMC9426443; doi:10.1128/msystems.00176-22)
Supplement: TEXT S1 [file msystems.00176-22-t0001.docx]

**Model Updates**

In order to utilize the iCre1355 model for this approach, we had to modify the way biomass and excretion were handled. Every biomass component was assigned a specific classification, as shown in Supplemental File 2. This split the biomass equation into ten sub-equations, where each sub-equation preserves the relative ratio among individual metabolites within the same classification. The sub-equations were then normalized by mass, so that 1 mmol of flux through a sub-equation produced 1 gram of mass imbalance. These normalized equations were added to the model.

We also added excretion equations for every single component within the biomass equation, so that the cell could dump components it was unable to catabolize. This meant that any biomass classification could be consumed for energy, as the steady-state limitation no longer applied; even if no degradation pathway exists for one metabolite in the sub-equation, it can be excreted while the rest are degraded for energy and/or carbon.
