# Supplementary material for: Rhythm of the Night (and Day): Predictive Metabolic Modeling of Diurnal Growth in Chlamydomonas
Source: mSystems. 2022 Jun 13;7(4):e00176-22. doi: 10.1128/msystems.00176-22 (PMC9426443; doi:10.1128/msystems.00176-22)
Supplement: TEXT S2 [file msystems.00176-22-t0002.docx]

**List of abbreviations**

AcCoA: Acetyl- Coenzyme A

AICc: Akaike Information Criterion

AKG: α-ketoglutarate

ATO1: Acetyl-CoA acyltransferase

CoA: Coenzyme A

CIT: citrate

DHAP: dihydroxyacetone phosphate

E4P: D-erythrose-4-phosphate

F6P: fructose 6-phosphate

FAP85: Flagellar associated protein

FBP: fructose biphosphate

FBA: Flux Balance Analysis

FEO: ferredoxin, oxidized

FER: ferredoxin, reduced

FoC: Ferrous cytochrome c

FUM: fumarate

GAP: glyceraldehyde 3-phosphate

G6P: glucose 6-phosphate

3GP: 3-phosphoglycerate

2PG: 2-phosphoglycerate

HPC: High performance computing

ICIT: isocitrate

LHSCR3.1: Light-harvesting complex stress-related protein 3.1, chloroplastic

MAL: malate

NGAM: non-growth associated maintenance

OA: oxaloacetate

PC1+: plastocyanin, reduced

PC2+, plastocyanin, oxidized

PEP: phosphoenolpyruvate

PYR: pyruvate

PQO: plastoquinone, oxidized

PQR: plastoquinone, reduced

QH2: Ubiquinone

RACK1: Receptor of activated protein C kinase 1

RBCS1: Ribulose bisphosphate carboxylase small subunit, chloroplastic 1

RNA: Ribonucleic acid

Ru5P: ribulose-5-phosphate

RuBisCo: Ribulose-1,5-bisphosphate carboxylase-oxygenase

SBP: sedoheptulose biphosphate

S7P: sedoheptulose 7-phosphate

SUCC: succinate

SUCCoA: Succinyl-coenzyme A

X5P: xylulose 5-phosphate

Z: photon.
