# Supplementary figures and images for: Rhythm of the Night (and Day): Predictive Metabolic Modeling of Diurnal Growth in Chlamydomonas
Source: mSystems. 2022 Jun 13;7(4):e00176-22. doi: 10.1128/msystems.00176-22 (PMC9426443; doi:10.1128/msystems.00176-22)

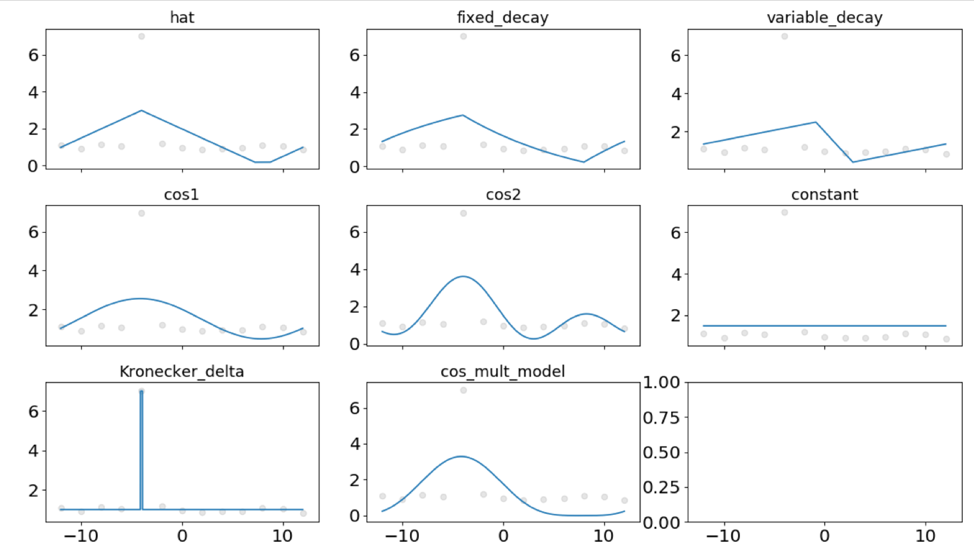

Supplement: FIG S1 [file msystems.00176-22-sf001.tif]
